# Adaptation of pine wood nematode *Bursaphelenchus xylophilus* to β-pinene stress

**DOI:** 10.1186/s12864-020-06876-5

**Published:** 2020-07-13

**Authors:** Yongxia Li, Yuqian Feng, Xuan Wang, Jing Cui, Xun Deng, Xingyao Zhang

**Affiliations:** 1 Lab. of Forest Pathogen Integrated Biology, Research institute of Forestry New Technology, Chinese Academy of Forestry, Beijing, 100091 China; 2 Co-Innovation Center for Sustainable Forestry in Southern China, College of Forestry, Nanjing Forestry University, Nanjing, 210037 China

**Keywords:** Pine wood nematode, β-Pinene, Reproduction rate, Mortality, Transcriptome

## Abstract

**Background:**

The pine wood nematode (PWN; *Bursaphelenchus xylophilus*) is the most damaging biological pest in pine forest ecosystems in China. However, the pathogenic mechanism remains unclear. Tracheid cavitation induced by excess metabolism of volatile terpenes is a typical characteristic of pine trees infected by *B. xylophilus*. β-pinene, one of the main volatile terpenes, influences PWN colonization and reproduction, stimulating pathogenicity during the early stages of infection. To elucidate the response mechanism of PWN to β-pinene, pathogenesis, mortality, and reproduction rate were investigated under different concentrations of β-pinene using a transcriptomics approach.

**Results:**

A low concentration of β-pinene (BL, C < 25.74 mg/ml) inhibited PWN reproduction, whereas a high concentration (BH, C > 128.7 mg/ml) promoted reproduction. Comparison of PWN expression profiles under low (BL, 21.66 mg/ml) and high (BH, 214.5 mg/ml) β-pinene concentrations at 48 h identified 659 and 418 differentially expressed genes (DEGs), respectively, compared with controls. Some key DEGs are potential regulators of β-pinene via detoxification metabolism (cytochrome P450, UDP-glucuronosyltransferases and short-chain dehydrogenases), ion channel/transporter activity (unc and ATP-binding cassette families), and nuclear receptor -related genes. Gene Ontology enrichment analysis of DEGs revealed metabolic processes as the most significant biological processes, and catalytic activity as the most significant molecular function for both BL and BH samples. Kyoto Encyclopedia of Genes and Genomes (KEGG) Orthology (KO) analysis showed that xenobiotics biodegradation and metabolism, carbohydrate metabolism, lipid metabolism, amino acid metabolism, metabolism of cofactors and vitamins, and transport and catabolism were the dominant terms in metabolism categories.

**Conclusion:**

In addition to detoxification via reduction/oxidation (redox) activity, PWN responds to β-pinene through amino acid metabolism, carbohydrate metabolism, and other pathways including growth regulation and epidermal protein changes to overcome β-pinene stress. This study lays a foundation for further exploring the pathogenic mechanism of PWN.

## Background

Pine wood nematode (PWN; *Bursaphelenchus xylophilus*) is a pathogen causing pine wilt disease (PWD), and infection can lead to tree death within 60–90 days of infestation, resulting in immense economic losses and ecological problems in regions where this pest species has been introduced. The pathogenic mechanism of PWD is complicated and involves many pathogenic factors including host pines, nematodes, beetles, fungi, bacteria, environmental factors, and other aspects [1, 2]. Three major hypotheses have been proposed for the pathogenic mechanism: the toxin theory, the cavitation theory, and the bacterial pathogenicity theory [3].

Once the above-ground parts of pine trees are infected with PWN and the organism is feeding on parenchymal cells, the terpene content is significantly increased in xylem, and vaporization of these substances can induce the formation of vacuoles in xylem that eventually cause tree death due to lack of water [4, 5]. Parenchyma cells of pines injured by the moving and feeding of nematodes synthesize terpenoids [6], including the monoterpenes alpha-pinene, camphene, beta-pinene, myrcene, limonene, beta-phellandrene, and p-cymene [7]. The major terpenes produced by pine trees are α-pinene and β-pinene, and both can enhance the resistance of pine trees to external biological stress from fungi, bacteria, insects, and nematodes [8–11]. These molecules exert strong poisoning and repelling effects against non-pine pests and pathogenic fungi [12–18]. Previous research indicated nematicidal activity of monoterpenoids against PWN [19–21]. Preliminary studies found that the ratio of α-pinene and β-pinene in healthy pines is 1:0.1, compared with 1:0.8 in infected trees [2, 10, 22]. Furthermore, propagation of PWN is significantly increased by high concentrations of α-pinene or β-pinene (275.2 mg/mL), suggesting that PWN may have the ability to utilize high concentrations of volatile terpenoids to overcome host defenses [10]. Previous studies have studied the effects of individual α-pinene on PWN mortality and reproduction rates and found that the PWN mortality increased with increasing concentrations of α-pinene, the reproduction rate reduced at 42.9 mg / mLα-pinene, and the reproduction rate increased at a higher concentration of 128.7 mg/mL [23]. Thus, studying interactions between β-pinene and PWN is important for understanding pathogenesis. Interestingly, low concentrations of β-pinene can inhibit the reproduction rate of PWN, while high concentrations can promote reproduction, suggesting that PWN may utilize high concentrations of β-pinene to promote reproduction and overcome resistance of host trees [10, 21, 24, 25].

In recent years, several PWN transcriptome studies have been performed. Kang et al. compared expressed sequence tags (ESTs) of *B. xylophilus* during dispersal and propagative stages [26]. Other studies analyzed PWN ESTs and derived genomic insight [27, 28], and Yan et al. compared transcriptome data from *B. xylophilus* and *Bursaphechus mucnatus* [29]. Li et al. explored the molecular response of PWN resistance to α-pinene through comparative transcriptomics of nematodes and found that the PWN genes involved in detoxification, transport, and receptor activity were differentially expressed [23]. Little is known about β-pinene metabolism in *B. xylophilus* and how this related to molecular pathogenicity. In particular, xenobiotic detoxification and pinene degradation processes in PWN have not been investigated, and neither has adaptation to parasitism. Investigating the transcriptomic responses of *B. xylophilus* to β-pinene stress could provide a better understanding of the biochemical and molecular processes involved in nematode development, reproduction, and host interactions.

In the present study, mortality and propagation of *B. xylophilus* were investigated using a differential transcriptomic RNA sequencing (RNA-Seq) approach following treatment with high and low concentrations of β-pinene via cotton ball bioassays. Functional genes related to detoxification were explored to elucidate the molecular mechanism of the PWN response to β-pinene stress, and provide a theoretical basis for controlling PWD.

## Results

### Effects of β-pinene concentration on viability and reproduction of *B. xylophilus*

**Fig. 1:**
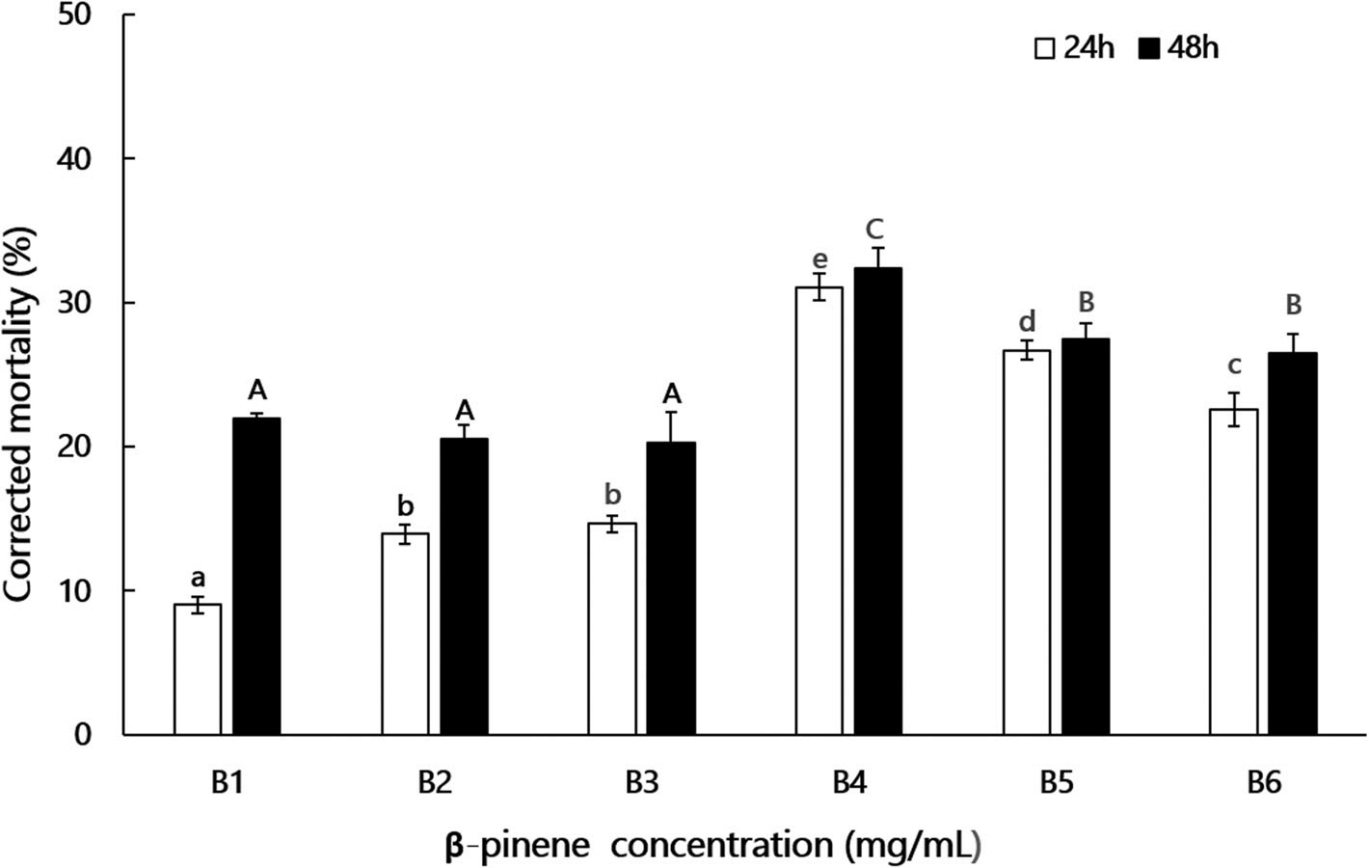
Corrected Mortality of PWN at different concentrations of β-pinene. B1, 4.29 mg/mL; B2, 17.16 mg/mL; B3, 25.74 mg/mL; B4, 42.9 mg/mL; B5, 128.7 mg/mL; B6, 214.5 mg/mL. For each treatment, the corrected mortality of PWNs exposed to β-pinene was corrected for the mortality of controls (CK, PWNs exposed to control using aqueous 0.5% Triton X-100 (m/m) solution). Data bars represent the mean of five independent replicates, and error bars represent standard errors of the mean. Different lowercase letters above bars indicate significant differences among the corrected mortality of PWNs exposed to β-pinene for 24 h (Tukey’s multiple comparison test, P<0.0.1), different capital letters above bars indicate significant differences among the corrected mortality of PWNs exposed to β-pinene for 48 h (Tukey’s multiple comparison test, P<0.01)

The propagation responses of PWN varied with the concentration of β-pinene; the *B. xylophilus* mortality rate differed significantly (24 h: F_5,29_ = 116.86, *P* < 0.01; 48 h: F_5,29_ = 11.795, *P* < 0.01), initially increasing then decreasing with increasing β-pinene concentration, peaking at 32.4% with 42.9 mg/mL β-pinene after 48 h (Fig. 1). Meanwhile, when the concentration of β-pinene was < 42.9 mg/mL, mortality decreased as the β-pinene concentration increased.

**Fig. 2:**
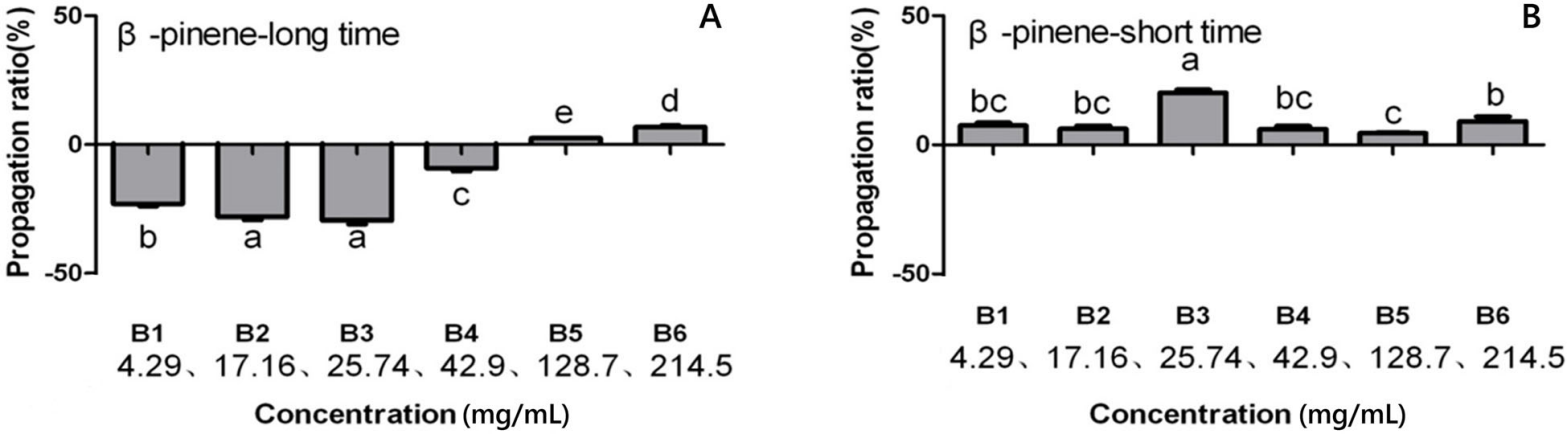
Effect of β-pinene concentration and treatment duration on PWN reproduction rate. **a**: Treatment for a long duration (7d), reproduction rate of pine wood nematodes under different β-pinene concentration for 7 days; **b**: Treatment for a short duration (48 h), reproduction rate of pine wood nematodes under different β-pinene concentration for 48 h. Data bars represent the mean of five independent replicates, and error bars represent standard errors of the mean. Different lowercase letters above or below bars indicate significant differences (Tukey’s multiple comparison test; *p* < 0.05)

The results indicate that β-pinene inhibited the propagation of *B. xylophilus*, and the effect was concentration-dependent (Fig. 2). When nematodes were treated with β-pinene for a short time (48 h), the propagation ratio was highest at a β-pinene concentration of 25.74 mg/mL. However, when nematodes were treated with β-pinene for a longer time (7 days), a β-pinene concentration < 42.9 mg/mL inhibited propagation, whereas a concentration > 128.7 mg/mL promoted propagation*.* Similar to the effects of α-pinene on PWN, 42.9 mg / mL and 128.7 mg / mL α-pinene is also the key concentration affecting the reproduction rate of PWM [23].

**Fig. 3:**
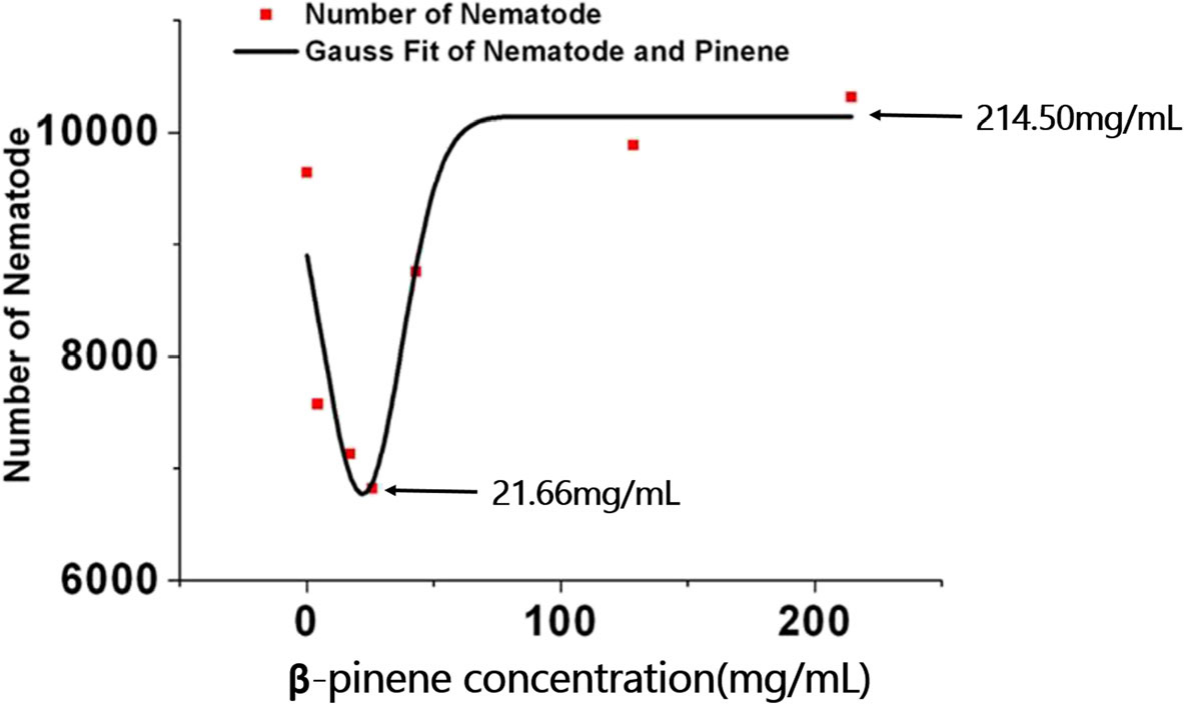
Effect of β-pinene concentration on PWN reproduction rate by curve fitting analysis. The line represents the best fit

Curve fitting analysis of the correlation between β-pinene concentration and propagation rate revealed an inflection point in the influence of β-pinene on propagation rate according to the equation y = − 0.007 × ^3^ + 2.6608 × ^2^ - 168.79 x + 3321.2. The lowest reproduction rate was observed at a β-pinene concentration of 21.66 mg/mL (Fig. 3).

### Overview of *B. xylophilus* transcriptome data

To further investigate the molecular response mechanism of PWN to β-pinene, cDNA libraries were generated by extracting total RNA from nematodes after culturing and treatment with low (BL, 21.66 mg/mL) and high (BH, 214.5 mg/mL) concentrations of β-pinene. Controls (CK) not receiving β-pinene were also included. After trimming adaptors, empty reads, and low-quality sequences, 2,592,186,600 bp and 2,378,778,000 bp of clean data were acquired for BL and BH, respectively, along with 2,121,994,600 bp of clean data for the CK group (Table 1). For the mixed stage cDNA library of *B. xylophilus*, 25,921,866 reads were obtained for BL sample, 23,787,780 reads were obtained for BH sample, and 21,219,946 reads were obtained for CK sample, with an average read length of 100 bp for all groups (Table 1). After assembly, ‘exon reads ≥5’ was applied as a threshold for significant gene expression. The number of genes expressed in the three treatment groups was 14,796 (CK), 15,159 (BL), and 15,062 (BH). Compared with the response of PWN to α-pinene, and the data and reads of the BL sample were lower than those (2,798,113,800 bp data and 27,981,138 reads) of the AD sample (PWN suffered low concentration 56.33 mg/mL α-pinene) while the BH sample were higher than those (2,267,290,600 bp data and 22,672,906 reads) of the AG sample (PWN suffered low concentration 214.5 mg/mL α-pinene), the number of genes expressed in the BH sample was slightly higher than that in the AG sample (14998) [30].

**Table 1:** Overview of transcriptome data from BL, BH, and CK groups

Sample	Data (bp)	Reads	Q20 (%)	Q30 (%)	Mapped (%)	ExpGene
CK	2,121,994,600	21,219,946	96.495	89.795	96.77	14,796
BL	2,592,186,600	25,921,866	96.640	90.125	96.97	15,159
BH	2,378,778,000	23,787,780	95.665	87.560	96.78	15,062

BL: PWN suffered 21.66 mg/mL β-pinene. BH: PWN suffered 214.5 mg/mL β-pinene. CK: Controls. ExpEene: Expression genes

**Fig. 4:**
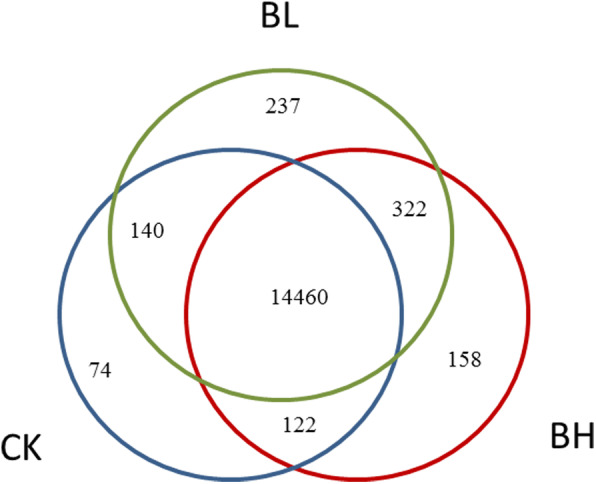
Venn diagram of differentially expressed genes (DEGs) from different treatments among the transcriptomes. BL, 21.66 mg/mL β-pinene. BH, 214.5 mg/mL β-pinene. CK, controls

By comparing the three treatment groups, 74 (CK), 322 (BL), and 158 (BG) genes were found to be specifically expressed between groups (Fig. 4). Compared with the response of PWN to α-pinene, number of DEGs in BL and BH samples were more than those in AD (121) and AG (97) samples, respectively [23]. However, this is only a qualitative analysis. Specific genes were not equal to genes with high expression and differential expression genes. Differentially expressed genes must be screened through calculation.

### Analysis of differentially expressed genes

Up- and downregulated DEGs were quantified and annotated in low and high β-pinene treatment groups. Compared with the control group, 659 and 418 DEGs were identified in BL and BH groups, respectively. 192 differential genes expressed in both high and low concentrations β-pinene conditions are identified and shown in Figure S1 (Fig. S1).

Functional analysis of the top ten upregulated DEGs identified oxidoreductase activity (CYP-33C2, CYP-33C4, CYP-33C9), membrane ion channel and transport (UNC-8), steroid hormone receptor activity (CBG01395), glycosyltransferase (ugt-48), and acid phosphatase activity (F21A3.11; Table 2). For the top ten downregulated DEGs, dehydrogenase/reductase (SDR-1), oxidoreductase (CYP-33E2), and ATP binding (F44E5.4) were identified (Table 2). According to the literature, the major classes of enzymes involved in detoxification are cytochromes P450 (CYPs), short-chain dehydrogenases (SDRs), UDP-glucuronosyl or glycosyl transferases (UGTs), and glutathione S-transferases (GSTs). Interestingly, when nematode worms were stressed by β-pinene, > 50% of detoxification genes were upregulated, while some detoxification genes were downregulated. These results suggest that different detoxification genes perform specific functions. In addition to detoxification genes, other redox-related genes such as T08H10.1 were upregulated following treatment with β-pinene. Transmembrane transporters such as F27D9.2 also appear to play an important role in the response to β-pinene. Under low concentrations of β-pinene, the product of this gene may form a channel that facilitates efficient detoxification. In addition, the top 20 genes of differential gene expression that are expressed only at high or low concentrations β-pinene are shown in Figure S2 (Fig. S2).

**Table 2:** The top ten DEGs up- and downregulated in *B. xylophilus* following exposure to β-pinene

Condition	ID	Fold Change	*P*-Value	Annotation
**CK-BL-up**	BUX.s00351.319	39.7037	0.0025	unc-8, membrane ion channel and transport
	BUX.s01144.121	29.7097	2.05E-15	CYP-33C4, oxidoreductase
	BUX.s00460.317	22.7929	2.63E-08	ugt-48, glycosyltransferase
	BUX.s00713.666	17.0264	1.97E-13	F21A3.11, acid phosphatase
	BUX.s00116.700	15.8343	1.25E-14	CYP-33C9, oxidoreductase
	BUX.s01063.115	15.7164	1.73E-15	CYP-33C2, oxidoreductase
	BUX.s00460.348	13.8545	6.80E-11	CBG01395, steroid hormone receptor
	BUX.s01198.204	11.8576	7.29E-10	T08H10.1, oxidoreductase
	BUX.s00460.319	11.0362	1.44E-10	ugt-48, glycosyltransferase
	BUX.s00460.315	10.0477	1.23E-11	ugt-48, glycosyltransferase
**CK-BL-down**	BUX.s01268.31	32.3698	4.57E-07	Alcohol dehydrogenase 1
	BUX.s00333.197	31.2817	0.0219	Alpha-(1,3)-fucosyltransferase C
	BUX.s00713.927	30.8087	5.47E-17	Dehydrogenase/reductase SDR-1
	BUX.s00116.698	25.2425	1.84E-17	CYP-33E2, oxidoreductase
	BUX.s01281.74	19.7473	1.54E-17	F44E5.4, ATP binding
	BUX.s00036.95	15.6409	0.0004	Strictosidine synthase family protein;
	BUX.s01143.144	15.3189	1.19E-14	T08H10.1, oxidoreductase
	BUX.c09083.1	15.1119	4.37E-05	GST-1, glutathione transferase
	BUX.s01518.87	14.4663	1.80E-05	Cystathionine gamma-lyase 2
	BUX.s00713.926	14.4408	2.31E-11	dhs-9, oxidoreductase activity
**CK-BH-up**	BUX.s00116.700	13.2573	3.42E-26	CYP-33C9, oxidoreductase
	BUX.s01063.115	9.8571	1.81E-24	CYP-33C2, oxidoreductase
	BUX.s01144.121	9.1615	4.47E-09	CYP-33C4, oxidoreductase
	BUX.s00460.348	9.0383	2.01E-12	CBG01395, steroid hormone receptor
	BUX.s01281.539	7.1376	0.0006	CBG06849, integral to membrane
	BUX.s01198.204	6.9227	1.27E-09	T08H10.1, oxidoreductase activity
	BUX.s01144.118	6.7539	8.33E-07	CYP-33C1, oxidoreductase
	BUX.s01092.48	6.1399	7.51E-12	CYP-32A1, oxidoreductase
	BUX.s00460.315	5.7481	7.06E-15	ugt-48, glycosyltransferase
	BUX.s01513.352	5.0654	0.0059	BEST-3, negative regulation of ion transport
**CK-BH-down**	BUX.s01513.34	29.6126	8.74E-03	17beta-hydroxysteroid dehydrogenase
	BUX.s01149.11	5.2565	5.46E-13	RHY-1, transferase
	BUX.s00961.158	4.1774	8.34E-12	HSP-70, ATP binding
	BUX.s01281.74	4.1731	9.26E-12	F44E5.4, ATP binding
	BUX.s01144.110	3.7650	6.35E-03	CYP-33E1, oxidoreductase
	BUX.s01211.14	3.7264	2.07E-09	C49F5.5, histone acetyltransferase
	BUX.s00139.27	3.3107	4.87E-07	Dehydrogenase/reductase SDR family member 1
	BUX.s00364.138	3.1628	1.24E-07	F27D9.2, transmembrane transport
	BUX.s00116.698	3.1201	3.55E-07	CYP-33E2, oxidoreductase
	BUX.s00364.193	3.1149	3.85E-08	Acyl-coenzyme A oxidase

CK-BL-up indicates up fold change in expression in BL samples compared with CK samples, CK-BL-down indicates down fold change in expression in BL samples compared with CK samples; CK-BH-up indicates up fold change in expression in BH samples compared with CK samples, CK-BH-down indicates down fold change in expression in BH samples compared with CK samples

### Analysis of functional genes in response to β-pinene stress

Based on the up- and downregulated DEGs identified from the transcriptome data, we evaluated functional PWN genes that might be involved in the response to stress induced by β-pinene. These genes included those involved in detoxification, ion channels and transporters, and receptors.

#### DEGs involved in detoxification metabolism

Comparison with the CK transcriptome revealed four categories of genes (CYPs, UGTs, SDRs, and GSTs) potentially involved in detoxification that were up- or downregulated. A total of 20 and 14 CYP450 genes, 13 and 13 UGT genes, 15 and 11 SDR genes, and four and one GST genes were differentially regulated in BL and BH samples, respectively (Table 3). Most UGTs were upregulated, whereas most GSTs and SDRs were downregulated, and CYPs were up- and downregulated in similar proportions (Table 3). CYP450s and UGTs play central roles in oxidative metabolism and detoxification. Cyp-33c2, cyp-33c4, cyp-33c9, and cyp-32a1 were significantly upregulated, while cyp-13a8 and cyp-33e2 were downregulated. Different CYP450 genes play different roles (Table 4). Four unigenes (cyp-33c2, cyp-33c4, cyp-33c9, and cyp-32a1) were upregulated in the PWN treated at low and high concentration α-pinene [23]. The number of CYPs differentially expressed in PWN treated with a low concentration of β-pinene was significantly higher than the number in the high concentration group.

**Table 3:** Detoxification-related genes up- and downregulated in *B. xylophilus* following exposure to β-pinene

Enzyme	Number of genes	
	CK-BL	CK-BH
	up/down	up/down
Total CYP450s	10/10	6/8
CYP-13A8	0/2	0/2
CYP-32A1	1/1	1/0
CYP-42A1	–	0/1
CYP-33C1	1/0	1/0
CYP-33C2	2/1	2/1
CYP-33C4	1/0	1/0
CYP-33C9	3/0	1/1
CYP-33E1	0/2	0/1
CYP-33E2	0/1	0/1
CYP450	2/3	0/1
Total UGTs	12/1	12/1
ugt-7	–	1/0
ugt-47	4/0	2/0
ugt-48	5/1	6/1
ugt-49	1/0	1/0
ugt-50	–	2/0
ugt-54	1/0	–
ugt-2a61	1/0	–
Total SDRs	3/12	3/8
sdr-1	0/5	1/4
sdr-4	0/2	0/2
sdr family	3/5	2/2
Total GSTs	0/4	0/1
GST-33	0/2	0/1
GST-39	0/1	–
GST-9	0/1	–

CK-BL indicates BL samples compared with CK samples, CK-BH indicates BH samples compared with CK samples. The same as below

**Table 4:** Summary of DEGs encoding detoxification, transport, and receptor proteins

classification	Genes	Function	Expression fold change	
			CK-BL	CK-BH
Detoxification	cyp-32a1	Monooxygenase activity	7.4417	6.1399
	cyp-13a8	Monooxygenase activity	0.4209	0.5703
	cyp-33c1	Monooxygenase activity	5.9065	6.539
	cyp-33c2	Monooxygenase activity	15.7164	9.8571
	cyp-33c4	Monooxygenase activity	29.7097	9.1615
	cyp-33c9	Monooxygenase activity	15.7164	13.2573
	cyp-33e2	Monooxygenase activity	0.0396	0.3205
	ugt-47	UDP-glucuronosyl transferase	4..3610	–
	ugt-48	UDP-glucuronosyl transferase	10.0477	5.7481
	ugt-49	UDP-glucuronosyl transferase	4.2132	1.6475
	sdr-1	Dehydrogenase/reductase	0.0940	0.3021
	sdr-4	Dehydrogenase/reductase	0.2308	0.5346
	dhs-2	Dehydrogenase/reductase	8.2780	3.2741
	dhs-27	Dehydrogenase/reductase	3.1062	2.7034
	gst-1	Glutathione S-transferase	0.0662	–
	gst-33	Glutathione S-transferase	0.1335	0.5688
	gst-39	Glutathione S-transferase	0.2232	–
Ion channels and transporters	unc-8	Ion channel and transport	39.7037	–
	unc-49	Channel activity	2.8943	–
	CBG06849	Integral to membrane	5.5478	7.1376
	CBG15937	Integral to membrane	4.1088	0
	T09B9.2	Transmembrane transport	2.4211	2.9392
	TWK-8	Potassium channel activity	3.0390	–
	ABC	ABC transporter	–	2.5745
	F44E7.7	Transmembrane transport	3.3631	–
	K09E9.1	Transmembrane transport	2.6022	2.2698
	Y19D10A.11	Transmembrane transport	0.2495	–
	F27D9.2	Transmembrane transport	0.1890	0.3162
	T19D12.9	Transmembrane transport	0.4394	–
Receptors	CBG01395	Steroid hormone receptor activity	13.8545	9.0383
	PTR-13	Hedgehog receptor activity	–	2.1015
	Exostosin-2	Multicellular organismal development	3.3019	–
	SREBP-1C	Sterol regulatory element-binding	4.7620	–
	START	START domain protein; cell division	2.7572	–
	shr-86	Steroid hormone receptor activity	5.7788	3.8288
	nhr-3	Nuclear receptor family member	2.2242	–
	nhr-10	Nuclear receptor family member	2.9472	2.6329
	nhr-31	Nuclear receptor family member	2.0544	–
	nhr-40	Nuclear receptor family member	2.7929	–
	nhr-62	Nuclear receptor family member	2.7607	2.4159
	nhr-70	Nuclear receptor family member	3.9372	2.5213

UGT-47 and UGT-48 were included among the differentially expressed UGTs. Although most SDR and GST genes were downregulated, some SDR genes such as dhs-2, dhs-27, and sdr-1 were upregulated more than 3-fold. These results suggest that CYPs, UGTs, and SDRs play an important role in detoxification, while GSTs do not appear to play a significant role in β-pinene induction.

#### DEGs related to ion channel and transporter activity

A large number of genes encoding channels and transporters were up- or downregulated in PWN treated with both high and low concentrations of β-pinene compared with controls. The number of upregulated and downregulated genes encoding channels and transporters was greater in BL samples than the BH group. In BL samples, unc-8 was upregulated > 39.7-fold compared with controls. Unc-8 is an important ion channel protein that can help to maintain osmotic pressure balance in cells [30]. Conversely, no significant difference was observed for unc-8 in BH or CK samples. Compared with the response of PWN to α-pinene, the fold change of unc-8 gene at low concentration of β-pinene was higher than that (23.1-fold) at low concentration α-pinene [23]. T09B9.2 was upregulated by 2.42-fold and 2.93-fold in BL and BH samples, respectively. T09B9.2 is an abundantly expressed member of the transporter superfamily and an important secondary transporter that spans the plasma membrane. In addition, genes encoding ATP-binding cassette (ABC) transporters were upregulated by 2.57-fold in BH compared with CK (Table 4). The fold change of ABC gene was lower than that PWN suffered low concentration α-pinene stress (4.55), and slightly higher than that of PWN under high concentration α-pinene stress (2.40) [23].

#### DEGs encoding receptors

PTR-13, a receptor involved in hedgehog signaling, was upregulated 2.10-fold in BH transcriptomes. CBG01395 is a receptor belonging to the nuclear hormone receptor family, and its expression was upregulated 13.85-fold and 9.03-fold in BL and BH samples. Exostosin-2 regulates soft tissue formation during growth, and thereby alters tissue architecture. The gene encoding exostosin-2 was upregulated 3.30-fold in BL. Nuclear receptors are primarily responsible for transmission of sterols, hormone signals, and other small molecules, hence regulating their expression can control the development, stability, and proliferation of cells [31–33]. Genes encoding nuclear receptors, including shr-86, nhr-3, nhr-10, nhr-31, nhr-40, nhr-62, and nhr-70, were upregulated in BL samples, while shr-86, nhr-10, nhr-62, and nhr-70 were also upregulated in BH samples (Table 3). The fold change of the genes encoding receptors (except PTR-13) responds to the low concentration condition was higher than that responds to the high concentration condition. Compared with the response of PWN to α-pinene, the fold change of DEGs encoding receptors were similar to that in response to β-pinene [23].

### GO enrichment analysis of DEGs

**Fig. 5:**
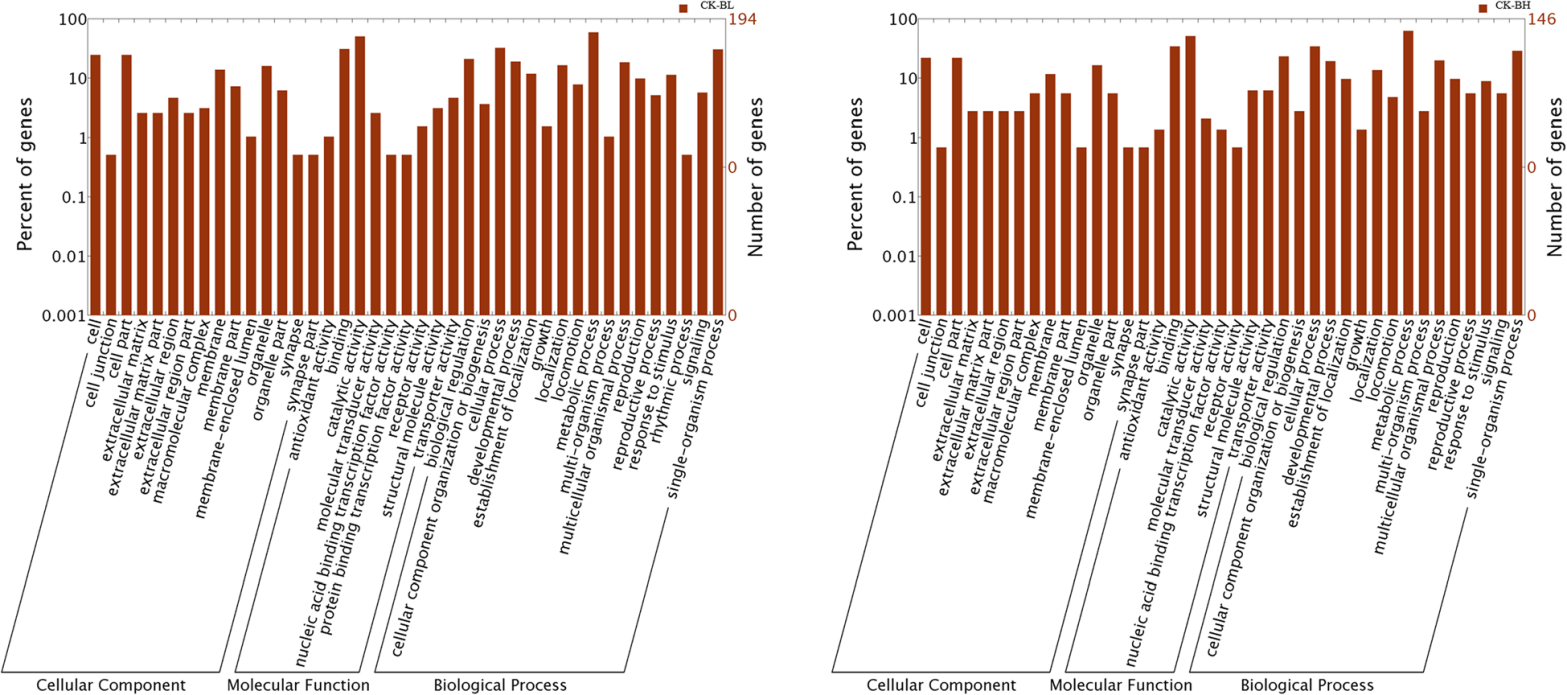
Functional classification and comparison of proteins encoded by DEGs based on GO annotation of BL and BH samples compared with CK samples. BL, 21.66 mg/mL β-pinene. BH, 214.5 mg/mL β-pinene. CK, controls. CK-BL indicates BL samples compared with CK samples, CK-BH indicates BH samples compared with CK samples

GO annotation was performed to functionally classify the identified proteins, which can provide details of the hierarchical relationships of cellular components, biological processes, and molecular functions (Fig. 5). GO annotation of DEGs between CK and BL samples found that 4.63% of DEGs in the cellular component category were associated with extracellular domain (GO:0005576) subcategories. For the molecular function category, 50.51% of DEGs were associated with catalytic activity (GO:0003824). For the biological process category, 59.27% of DEGs were linked to metabolic processes (GO:0008152).

GO annotation of DEGs between CK and BL samples found that 2.7% of DEGs in the cellular component category were related to the extracellular domain (GO:0005576) subcategory. For the molecular function category, 51.36% of DEGs were associated with catalytic activity (GO:0003824), and 16.43% with redox enzyme activity (GO:0016491). For the biological process category, 63.01% of DEGs were linked to metabolic processes (GO:0008152), and 27.39% were involved in single-organism metabolic processes (GO: 0044710). Cyp-13a8 and cyp-33c9 are linked with catalytic activity (GO:0003824), metabolic processes (GO:0008152), redox enzyme activity (GO:0016491), and single-organism metabolic processes (GO: 0044710). Cyp-33c1 and cyp-33c4 are involved in the regulation of growth (GO:0040008), positive regulation of growth (GO:0045927), regulation of growth rate (GO:0040009), positive regulation of growth rate (GO:0040010), positive regulation of the biological process (GO:0048518), regulation of the biological process (GO:0050789), and biological regulation (GO:0065007). Genes involved in the biological process (GO:0008150) included cyp-13a8, cyp-33c1, cyp-33c4, cyp-33c9, unc-8, TWK-8, nhr-3, nhr-62, and nhr-10. Genes involved in binding (GO:0005488) and molecular function (GO:0003674) included cyp-33c9, gst-39, nhr-3, nhr-40, nhr-62, and nhr-10.

### KEGG functional annotation of DEGs

**Fig. 6:**
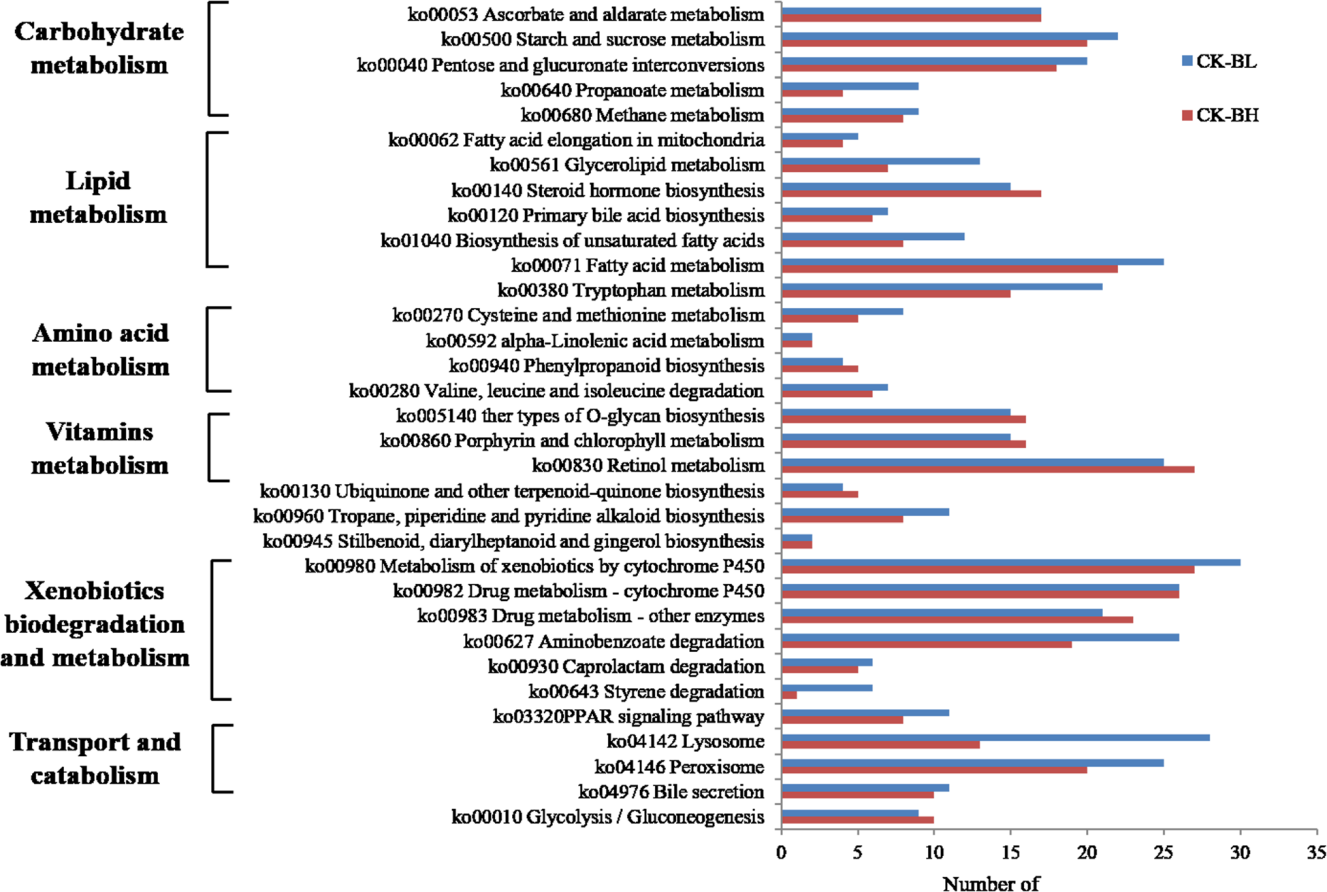
Major KEGG metabolic pathways altered in PWN following exposure to β-pinene

For functional annotation, open reading frame (ORF) sequences of *B. xylophilus* were mapped to reference canonical pathways in the KEGG database. A total of 318 (BL) and 266 (BH) sequences were mapped to 37 and 31 KEGG pathways, respectively (Fig. 6). KEGG Orthology (KO) analysis showed that xenobiotics biodegradation and metabolism, carbohydrate metabolism, lipid metabolism, amino acid metabolism, and metabolism of cofactors and vitamins were the dominant terms in the metabolism categories. Furthermore, transport and catabolism appear to play an important role in cellular processes. Metabolic pathways related to the immune system, digestive system, and endocrine system (within organismal systems) were significantly enriched in both treatment groups. KEGG functional annotation of the two transcriptomes showed that enzymes involved in xenobiotic degradation and metabolism were abundant, accounting for half of all DEGs involved in these metabolism pathways (115 and 113 DEGs for BL and BH, respectively) (Table 5). Thus, these metabolic pathways likely play an important role in the responses of PWN to β-pinene stress. Those were similar to the major metabolic pathways in response to α-pinene stress, except that the number of genes involved in different metabolic pathways is slightly different [23].

**Table 5:** Top 13 pathways involving DEGs identified in BL and BH samples

KEGG (KO) term	Number of genes	
	CK-BL	CK-BH
**Metabolism**		
Carbohydrate metabolism	68	65
Energy metabolism	9	9
Lipid metabolism	80	57
Amino acid metabolism	42	26
Glycan biosynthesis and metabolism	15	16
Metabolism of cofactors and vitamins	40	48
Metabolism of terpenoids and polyketides	0	3
Biosynthesis of other secondary metabolites	13	10
Xenobiotics biodegradation and metabolism	115	113
**Cellular processes**		
Transport and catabolism	53	20
**Organismal systems**		
Immune system	20	
Digestive system	11	10
Endocrine system	11	8

Genes enriched in xenobiotics biodegradation and metabolism under low concentrations of β-pinene were significantly greater in number than those under high concentrations (Fig. 6). These DEGs were mainly associated with metabolism of xenobiotics by cytochromeP450 (ko00980), aminobenzoate degradation (ko00627), drug metabolism-cytochromeP450 (ko00982), and drug metabolism of other enzymes (ko00983) dominated by cyp450s. The major genes involved in these pathways were ugt-47, ugt-48, ugt-49, and gst-39. These KEGG pathways belong to the xenobiotics biodegradation and metabolism category. In addition to genes enriched in vitamin metabolism, the number of genes enriched in carbohydrate metabolism, amino acid metabolism, transport and catabolism, digestive system, and immune system categories by a low concentration of β-pinene was greater than that induced by a high concentration of β-pinene. T09B9.2, an ABC transporter, and Y19D10A.11 are involved in lysosomal (ko04142) function, while sdr-4 and dhs-28 are involved in peroxisome (ko04146) function, and these KEGG pathways belong to the transport and catabolism category. A greater number of DEGs related to steroid hormone biosynthesis and glycolysis/gluconeogenesis were induced by a high concentration of β-pinene than a low concentration. Interestingly, ugt-47, ugt-48, and ugt-49 are not only involved in steroid hormone biosynthesis (ko00140) and the metabolism of lipids, but also play a role in ascorbate and aldarate metabolism (ko00053), pentose and glucuronate interconversion (ko00040), starch and sucrose metabolism (ko00500), other types of O-glycan biosynthesis (ko00514), porphyrin and chlorophyll metabolism (ko00860), and retinol metabolism (ko00830) KEGG pathways.

### Verification of RNA-Seq data by qRT-PCR

**Fig. 7:**
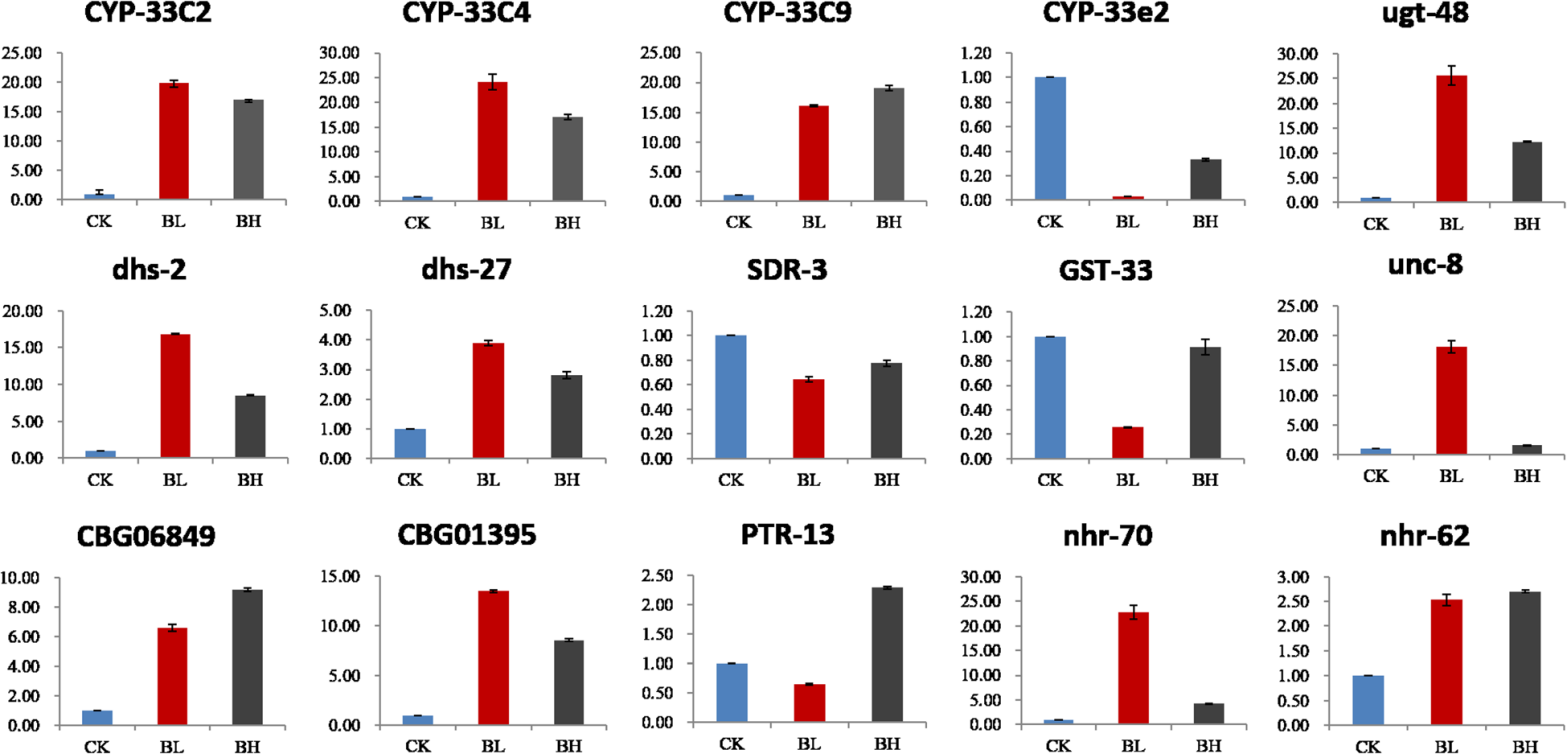
Verification of selected DEGs identified by RNA-Seq using qRT-PCR. BL, 21.66 mg/mL β-pinene. BH, 214.5 mg/mL β-pinene. CK, controls. The qRT-PCR data are the average of triplicate experiments, and bars represent standard error. The ordinate represents relative gene expression levels

To verify the changes in expression levels induced by low or high concentrations of β-pinene measured by transcriptome analysis, 15 potentially key genes (CYP-33C4, CYP-33C2, CYP-33C9, CYP-33e2, ugt-48, DHS-2, DHS-27, SDR-3, GST-33, unc-8, CBG06849, CBG01395, PTR-13, nhr-70, and nhr-62) were selected for verification by qRT-PCR. The expression profiles of genes obtained by qRT-PCR were very similar to those obtained by RNA-Seq, consistent with the GO and KEGG functional annotation of DEGs (Fig. 7).

## Discussion

*B. xylophilus* is one of the most damaging forest pests, but its pathogenic mechanism remains poorly understood. Research has shown that pines release volatile terpenoids to resist invasion by PWN [22, 34]. Massive accumulation of terpenes in xylem [5] promotes the vaporization of tracheids and the formation of voids in xylem [4], eventually resulting in tree death due to moisture loss. Therefore, there is a complex interaction between pine trees, terpenoid metabolism, and PWN.

Different concentrations of β-pinene have different effects on the reproductive rate of *B. xylophilus.* Mortality of PWN is highest at a β-pinene concentration of 20 mg/mL [20]. β-pinene can both promote and inhibit the reproduction of *B. xylophilus* [35]. For example, propagation of PWN was significantly stimulated at a high (275.2 mg/mL) concentration of β-pinene [10]. The results of the present study showed that a low concentration of β-pinene inhibits PWN reproduction, whereas a high concentration promoted reproduction. A concentration of 214.5 mg/mL β-pineneresulted in the fastest rate of PWN reproduction, consistent with previous studies [10]. Our research found that high concentrations (BH, C>128.7 mg/mL) promoted the reproduction of pine wood nematodes, and low concentrations (BL, C<25.74 mg/mL) inhibited the reproduction of pine wood nematodes. This result is consistent with the results of previous studies on the resistance of pine wood nematodes to different concentrations α-pinene [23]. The substances released by the pine’s defense response are complex, and the next study should cover the combined effects of β-pinene and other components on PWN. Simultaneously, under high-concentration β-pinene conditions, the expression changes of most pine wood nematodes gene involved in detoxification were less than that under low-concentration conditions. Previous reports have indicated that *Serratia marina* LCN16, pinewood nematode-associated bacteria, has many common characteristics with endophytes (plant-associated bacteria), such as genes coding aromatic compound degradation and detoxifying enzymes [36, 37]. Therefore, it is speculated that the high concentration of β-pinene induced by the pine defense response may induce the ability of PWN-associated bacteria to degrade and detoxify β-pinene, which may assist the PWN to resist defensive response of the pine trees, and promote reproduction ability of the PWN’s population. Future research needs to focus on the interaction among pine wood nematode-associated bacteria-pine resistance-pine wood nematodes.

PWN expresses a large number of detoxification- and reproduction-related genes involved in the response to β-pinene stress. Following infestation of pine trees, PWN secretes detoxification proteins that combine redox and other effects to endow resistance to poisoning by host terpenes, thereby assisting colonization and prolonging disease [5, 28, 29, 38]. PWN resistance to β-pinene therefore involves a complex multi-gene interaction process [28, 39, 40]. In the present study, we identified numerous putative detoxification-related proteins that were upregulated in PWN following exposure to β-pinene, including cytochrome P450s (CYPs), short-chain dehydrogenase (SDRs), glucuronate dehydrogenases (UGTs), ATP-binding cassette (ABC) transporters, and various ion channel and transporter-related proteins. Heme-containing CYP-450 proteins are a ubiquitous family of monooxygenase isozymes responsible for the oxidative metabolism of a wide variety of endogenous and exogenous substrates [41]. SDRs are also important for detoxification metabolism in nematodes, especially in the early stages of the detoxification process [29, 42].GSTs are multifunctional proteins that are essential for xenobiotic metabolism as well as detoxification of endogenous compounds, and they provide protection against peroxidative damage**.** UGTs and GSTs are the main enzymes mediating phase 2 metabolism in *C. elegans*, in which the actual detoxification reactions of xenobiotic metabolism take place [29, 42]. The study found that the expression level of GST gene in PWN treated with high-concentration β-pinene was higher than that in PWN treated with low concentration, and both were lower than the control. GST was expressed in the dorsal gland cell of PWN after infection of the host or only expressed at 15 and 6 dpi, which could be delivered into the host protect PWN from host defence responses during phytophagy [43]. GSTs secreted by PWN into the host participate in the detoxification of host-derived defence compounds and enable successful parasitism [44]. Based on the known functions of these proteins and our current results, these genes may perform key detoxifying roles during the first and second stages of xenobiotic metabolism leading to resistance to β-pinene, consistent with previous results on *C. elegans* [42].

ABC family members and other channels and transporters mediate the final stages of detoxification metabolism [42]. ABC transporters are transmembrane proteins that transport various substrates across extra- and intracellular membranes, including metabolic products, lipids and sterols, and drugs and their metabolites. Some ABC transporter genes are upregulated in PWN after inoculation on *Pinus thunbergii*, and these genes contribute to the degradation of host cells and play a detoxifying role against terpenes [45]. A total of 60 ABC transporter genes are present in the *C. elegans* genome that may be involved in detoxification metabolism [46].

Unc-8 is an important channel protein that helps to maintain osmotic pressure balance in cells, and acts as an ion channel in *C. elegans* [30]. Unc-8 plays an important role in the transport of modified toxic substances and the maintenance of ion balance weight. T09B9.2, belonging to the major facilitator transporter superfamily [47], is an important secondary transport protein mediating transport through the cytoplasm and endometrium. In the present study, expression of ABC transporter genes was increased by 4.56- and 2.57-fold following treatment with β-pinene at low and high concentrations, respectively. Unc-8 was upregulated 29.71-fold at low β-pinene concentrations, while T09B9.2 expression was increased 2.42- and 2.15-fold following treatment with β-pinene at low and high concentrations, respectively. Meanwhile, various channels and transporters, such as F27D9.2, T19D12.9, and Y19D10a.11, were downregulated. These results suggest that ABC, Unc-8, T09B9.2, and other genes may play key roles in the transport of metabolites related to β-pinene. Some ion channel genes were downregulated; this may be necessary to ensure efficient detoxification and transport.

In addition to detoxifying- and transporter-related genes, expression of hormones related to physiological growth and other stress response proteins was also altered following treatment with terpenes, indicating further changes in physiological activities that take place in PWN under adverse conditions.

The hedgehog (Hh) signaling pathway plays a key role in embryonic development, regulating cell proliferation and differentiation, and coordinating tissue and organ development. As an evolutionarily conserved signaling pathway, it plays an important regulatory role in the development of vertebrates and invertebrates [48, 49]. PTR (Patched-related protein) is a receptor protein with hedgehog activity. There are two types of PTC (Patched) and 24 types of PTC -related (PTR) proteins in *C. elegans*, and PTC-1 is essential for the embryo cytoplasmic division and germ cell growth [50].

Protein CBG01395, belonging to the nuclear hormone receptor family, is a steroid hormone receptor in cells that mediates steroid hormone signal transduction via specific gene expression over several hours or a few days, thereby altering physiology and regulating heat shock protein expression [51]. Nuclear receptors are mainly responsible for the transmission of sterols, hormone signals, and other small molecules that regulate the expression of specific genes controlling cell development, stability, and metabolism [31–33]. In the present study, compared with the control group, expression of PTR-13 was upregulated 2.10-fold following treatment with high concentrations of β-pinene, while CBG01395 was upregulated 13.85-and 9.04-fold, respectively. Expression of multiple nuclear receptors was upregulated, especially at low concentrations of β-pinene, presumably to regulate downstream genes. In addition, exostoses 2, a stress response protein regulating the formation of soft tissue during growth that can lead to tissue abnormalities, was upregulated 3.30-fold in the presence of low concentrations of β-pinene. Meanwhile, acetylcholine receptor subunit alpha-type deg-3, a cell growth-regulating nucleolar protein, sterol regulatory element-binding protein 1, and various other proteins were also upregulated following β-pinene stress. These results indicate that PWN may detoxify substances through redox reactions. Some hormone receptors related to growth were also upregulated, presumably to ensure normal physiological activities in PWN under adverse conditions. Nuclear receptors are known to regulate multiple genes in response to β-pinene stress, and the synergistic action of multiple genes likely assists PWN resistance to β-pinene.

Furthermore, the results of KEGG pathway and GO annotation analyses showed that CYP genes differentially regulated in PWN following β-pinene stress are mainly involved in REDOX metabolism and growth, while UGT genes are mainly involved in detoxification pathways and carbohydrate metabolism, and UNC genes and nuclear receptor genes are mainly associated with binding, biological process, and molecular function categories. ABC transporter genes encode proteins responsible for the transport of substances in lysosomes, and many are involved in single-organism metabolic processes. The DEGs identified in the present study are thought to participate in the metabolism of β-pinene. However, they may not constitute complete pathways for β-pinene metabolism, and some other mechanisms and/or pathways may be present in PWN. It may therefore be worth investigating this matter further.

## Conclusions

In this study, we report on the effects of different concentrations of β-pinene on the mortality and reproduction rate of PWN in vitro. The response mechanism of PWN to β-pinene was examined via comparative transcriptomics of PWN. Based on all available data, we conclude that a low concentration of β-pinene can inhibit reproduction of PWN, whereas a high concentration can promote reproduction. The mechanism of the response of *B. xylophilus* to β-pinene stress appears to involve detoxification followed by excretion of metabolites by ion and small molecule transporters. Some epidermal protein synthesis genes were found to be upregulated, presumably to help the organism cope with injuries caused by β-pinene stress. Upregulation of hormone receptors and transcription-related factors may help to ensure normal physiological activities in PWN in the face of host defenses. The metabolism of PWN in the presence of low concentrations of β-pinene was stimulated more than in the presence of high concentrations, possibly because low concentrations of β-pinene may cause greater stress, promoting larger changes in gene expression. In addition to detoxification of β-pinene via redox reactions, transport, immune, and digestive systems also appear to play important roles in PWN exposed to low concentrations of β-pinene. In addition to redox-based detoxification, responses to β-pinene include changes in amino acid metabolism, carbohydrate metabolism, and other pathways including growth regulation and epidermal proteins changes, all of which work in concert to overcome β-pinene stress. This study is conducive to exploring the molecular mechanism of PWN response to monoterpenes and the pathogenesis of PWN. In future studies, more detailed functional analysis of the key genes identified in this study should be carried out to unravel the complex mechanisms governing the responses to β-pinene stress in this economically important pest species of pine forests.

## Methods

### Strain and cultivation of PWN

The Nxy61 strain of PWN was used for transcriptome analysis. All nematodes were isolated from pinewood chips infested with *Pinus. massoniana* in Ningbo, Zhejiang Province, China, and cultured in our laboratory. Nematodes were cultured on fungal mats of *Botrytis cinerea* grown on potato dextrose agar (PDA) plates at 25 °C for 7–10 days, and then separated using the Baermann funnel technique [52]. After collection, light microscopy, and purification, nematodes were cultured on corn-meal agar (CMA) with *B. cinerea* until the plate was covered by PWN, and the plate was stored in a refrigerator at 4 °C for long-term preservation.

A 50 μL sample of nematode liquid (~ 3000 nematodes including eggs, 2–4 juvenile stages, and adults) was inoculated and cultured on fungal mats of *B. cinerea* (90 mm) growing on PDA medium (Nissui-seiyaku, Tokyo, Japan) containing 100 mg/mL streptomycin and 25 mg/mL chloramphenicol at 25 °C for 7 days. Nematodes were extracted for 4 h using the Baermann funnel technique. Nematode liquid was washed twice with 1× Phosphate Buffered Saline Tween-20 (PBST) by centrifugation at 8000×*g* for 5 min each time to remove impurities. Nematode liquid was diluted with 1 × PBST and sterilized by adding 100 μg/mL streptomycin and 20 μg/mL chloramphenicol. The mixture was incubated for 8 h, washed twice with 1× PBST by centrifugation at 8000×*g* for 5 min each time to remove supernatant, and stored in a refrigerator at 4 °C for later use.

### Mortality and propagation of PWN induced by β-pinene

#### Mortality

Six different concentrations of β-pinene (4.29, 17.16, 25.74, 42.9, 128.7, and 214.5 mg/mL) were prepared using 0.5% (m/m) Triton X-100 [21]. Next, 200 μL samples of different concentrations of β-pinene solution and 50 μL of PWN mixture (~ 500 nematodes, juveniles, and adults) were mixed together in a 96-well plate (Falcon, Franklin Lakes, NJ, USA), and ~ 500 nematodes were treated with 0.5% Triton X-100 (m/m) solution as controls. After incubating for 24 or 48 h at 25 °C in darkness with shaking, the 96-well plate was placed in a shaking incubator at 150 rpm for 30 min. PWN mortality (immobility) was assessed under an optical microscope. Each treatment included five replicates.

#### Propagation of PWN

For short-term treatment, the effect of β-pinene on the reproduction rate of PWN was determined by the soaking culture method. PWN were treated with six concentrations of β-pinene solution for 48 h, centrifuged, separated, and diluted. A 50 μL sample of PWN suspension (~ 6000 nematodes) was injected, and samples were cultivated on potato dextrose agar (PDA) with *B. cinerea* in a 35 mm diameter culture dish sealed with parafilm (Pechiney Plastic Packaging, Menasha, WI, USA) and incubated in the dark at 25 °C for seven days. Controls were treated with sterile water. Living nematodes were isolated using the Baermann funnel technique and counted. Each treatment included five replicates. For long-term treatment, cotton ball bioassays were used to determine whether β-pinene inhibited or stimulated PWN propagation (Kong et al., 2007). A hole (~ 5 mm) was drilled at the center of a PDA plate (35 mm diameter) containing *B. cinerea*, and a cotton ball (~ 5 mm diameter) treated with 50 μL of one of the six different concentrations of β-pinene was placed over the hole. A 50 μL sample of nematode suspension (~ 6000 nematodes) was then injected into the fungal plate. Controls were injected with 50 μL of 0.5% Triton X-100 (m/m) solution. Both treatment and control plates were sealed with parafilm and incubated at 25 °C in the dark for 7 days. Living nematodes were isolated using the Baermann funnel technique and counted. Each treatment included five replicates.

### Preparation of PWN for transcriptome analysis

Based on the physiological measurement results, 21.66 mg/mL (BL) and 214.5 mg/mL (BH) β-pinene were chosen as low and high concentrations for treating nematodes. BL or BH in 0.5% Triton x-100 were added to nematode solutions, 0.5% Triton X-100 (m/m) alone was added to controls, and samples were subjected to gentle shaking at 25 °C for 48 h, and then collected by centrifugation at 5000×*g* for 5 min. Nematodes were washed five or six times with PBST and then separated into RNAse-free 1.5 mL centrifuge tubes. Each centrifuge tube containing ~ 20,000 PWN was stored at − 80 °C and used for RNA extraction and cDNA synthesis as described in the next section. Each treatment included three replicates.

### Total RNA extraction and cDNA library construction

Total RNA from *B. xylophilus* treated with or without β-pinene as described in the previous section was extracted with an RNAprep Pure Tissue kit (Tiangen Biotech, Beijing, China) according to the manufacturer’s protocol. Extracted RNA was checked using a Nanodrop ND-8000 instrument, and the absorbance ratio at 260/280 nm (OD260/280) was recorded. An Agilent 2100 Bioanalyzer (Agilent, Mainz, Germany) was used for RNA qualitative and quantitative analysis. RNA samples were stored at − 80 °C until needed. Total RNA was digested with RNase-free DNase I at 37 °C for 30 min to remove contaminating genomic DNA, and samples were prepared using an Illumina kit (Illumina, San Diego, CA) according to the manufacturer’s protocol. First, mRNA was separated from total RNA using oligo-dT-adsorbed magnetic beads, and cleaved RNA fragments were used for first- and second-strand cDNA synthesis using reverse transcriptase and random hexamer primers. Products were amplified by PCR to generate the final cDNA libraries containing fragments ~ 200 bp in size, which were sequenced from both ends by an Illumina Genome Analyzer at Beijing Genomics Institute, Shenzhen, China.

### Transcriptome data analysis

The WapRNA RNA sequence workstation (http://waprna.big.ac.cn/rnaseq) was used to carry out transcriptome analysis [53]. Raw cDNA sequences were cleaned by removing adaptor, polyA, and low-quality sequences. High-quality EST sequences were assembled into contigs using Trinity (http://trinityrnaseq.sourceforge.net). Contigs longer than 300 bp were used for analysis. Sequencing data were compared with the UniProtKB protein database (http://web.expasy.org) for functional annotation, and with the pine wood nematocyst genome database using Tophat2 software (ftp://ftp.sanger.ac.uk/pub4/pathogens/Bursaphelenchus/xylophilus/Assembly-v) [54].

Gene expression data were mapped with unique mapped reads using the following equation:

$$ RPKM=\frac{exon_{\mathrm{reads}\kern0.5em }\times {10}^9}{{\mathrm{unique}}_{\mathrm{reads}}\kern0.5em \times {Gene}_{length}} $$

The DEGseq method [55] was used to identify differentially expressed genes (DEGs) under different treatments (*p* < 0.05). The dataset was BLAST searched against the NCBI non-redundant (nr) database (http://www.ncbi.nlm.nih.gov) and the UniProt database for Gene Ontology (GO) annotation using Blast2GO [56], and against the Kyoto Encyclopedia of Genes and Genomes (KEGG) database for homology analysis (E-value ≤1e^− 5^) [57]. Pearson’s correlation coefficients were used to evaluate the proportion of transcripts linked to each GO term and KEGG biological pathway for all three transcriptomes.

### Identification of statistically enriched GO terms and KEGG pathways

The hypergeometric test was used to measure significantly enriched GO terms in target gene groups compared with controls [58, 59] using the following formula:

$$ p=1-{\sum}_{i=0}^{m-1}\frac{\left(\begin{array}{c}M\\ {}i\end{array}\right)\left(\begin{array}{c}N-M\\ {}n-i\end{array}\right)}{\left(\begin{array}{c}N\\ {}n\end{array}\right)}, $$

where N is the number of genes with a GO annotation, n is the number of DEGs in N, M is the number of genes annotated to a certain GO term, and m is the number of DEGs in M. GO terms with a *p*-value cut-off of 0.005 were deemed to be enriched. In addition, to identify enriched pathways, the hypergeometric test was used in a similar manner to measure the relative coverage of annotated KEGG orthologous groups of pathways in the background, and pathways with a *p*-value cut-off of 0.005 were considered enriched [60].

### Verification of RNA-Seq data by quantitative real-time PCR (qRT-PCR)

Identical amounts of each RNA sample were used for qRT-PCR and transcriptome sequencing. Primer pairs for candidate genes were designed using OligoArchitect (http://www.oligoarchitect.com; Supplementary Table S1). RT-qPCR was performed on an ABI 7500 Real-Time PCR system (Applied Biosystems, Forster City, CA, USA) with 25 μL reactions containing cDNA template, primers, and SYBR Premix Ex Taq Mix (Takara Co., Otsu, Japan) and ROX Reference Dye II (Takara Biotechnology, Co., Ltd). Cycling parameters included an initial denaturation at 95 °C for 60 s, followed by 40 cycles at 95 °C for 15 s and 60 °C for 35 s. The actin gene of *B. xylophilus* was used as an internal control to normalize gene expression levels. Relative expression levels of DEGs were calculated using the 2^-ΔΔCT^ method [61], and each treatment included three replicates.

### Statistical analysis

Data were statistically analyzed using Origin 8.0 (OriginLab Corporation, Northampton, MA, USA) and SPSS 17.0 for Windows (SPSS Inc., Chicago, IL, USA). Relative expression levels of the same gene in different groups were analyzed by analysis of variance (ANOVA) and means were separated with Tukey’s multiple comparison tests.

## Supplementary information

**Additional file 1: Table S1**. Primers used in real-time PCR verification of RNA-Seq data.

**Additional file 2: Figure S1**. Heat map of the amplitude of 192 differentially expressed genes expressed in pine wood nematodes at high and low concentrations β-pinene conditions.

**Additional file 3: Figure S2**. Heat maps of the top 20 differentially expressed genes for pine wood nematodes with high or low concentrations β-pinene treatment.

## Data Availability

All data generated or analysed during this study are included in this published article. RNA sequencing data for the experiments have been deposited to the NCBI Sequence Read Archive (SRA) –BioProject accession number PRJNA640733.

## Abbreviations

KEGG: Kyoto Encyclopedia of Genes and Genomes
KO: Kyoto Encyclopedia of Genes and Genomes Orthology
CYPs: Cytochromes P450s
SDRs: Short-chain dehydrogenases
UGTs: UDP-glucuronosyl or glycosyl transferases
GSTs: Glutathione S-transferases
DEGs: Differentially expressed genes
ESTs: Expressed sequence tags
ORF: Open reading frame
ABC: ATP-binding cassette transporter
