# Supplementary material for: Adaptation of pine wood nematode Bursaphelenchus xylophilus to β-pinene stress
Source: BMC Genomics. 2020 Jul 13;21:478. doi: 10.1186/s12864-020-06876-5 (PMC7358211; doi:10.1186/s12864-020-06876-5)
Supplement: Supplementary file 1 — Additional file 1: Table S1. Primers used in real-time PCR verification of RNA-Seq data. [file 12864_2020_6876_MOESM1_ESM.docx]

**Table S1 Primers used in real-time PCR verification of RNA-Seq data**

| **Gene name** | **Forward primer** | **Reverse primer** |
| --- | --- | --- |
| CYP-33C4 | GATACAGATACAGCAAGGAGAAT | AACAACCAGACGGAGGAA |
| CYP-33C2 | CAGTCTGGTTAATGAAGTT | ATAGCCGTTGAGATTGTA |
| CYP-33C9 | CTGATATTGTGGCTTGCTA | AGTCCTACGAGTTGTTGA |
| CYP-33e2 | GGCCCTCGGATACCTTGTTC | AGATTTCCATCCGAGCCAGC |
| UGT-48 | GATGAGATGATGGACAATGGT | TTAGTTGAGATTCGCAAGCATA |
| DHS-2 | CGGTATTGCTGCTATTGG | AAGGAGAGGAATGAGAAGAT |
| DHS-27 | AGTTCCTGGATGACATTA | ATAGTACGCTACAGAAGT |
| SDR-3 | TATGAAGGTAATTGAAAGG | ATTATTGACCGAATGAAG |
| GST-33 | GATGATGGTAACGGAGATA | TCCAAGTTATTCAGCAAGA |
| UNC-8 | GGAGATGTGGTCAACTTC | CAGAGGCACTAAGATTCG |
| CBG06849 | ACCTCTACAGTGACATTATG | CTTGGAAATTGCGATGAC |
| CBG01395 | TGGTGGTTCATTATGGCTAT | CGGTGTCATTAGGTGTAGAT |
| PTR-13 | AAAGTTGTTGGATGAAGC | CATTCTCCTTGATATGGC |
| NHR-70 | CGTCTTCTCCTTCTCAACT | GATGGTCGTAGCAGTGTT |
| NHR-62 | TACAACAACCTACATAACC | GCGACGAGATATAATACAT |
| Reference gene | TCCGTACCCTGAAGTTGGCTAA  CC | AAGTGGAGACGAGGGAATGGAA  CC |
