# Supplementary figures and images for: Adaptation of pine wood nematode Bursaphelenchus xylophilus to β-pinene stress
Source: BMC Genomics. 2020 Jul 13;21:478. doi: 10.1186/s12864-020-06876-5 (PMC7358211; doi:10.1186/s12864-020-06876-5)

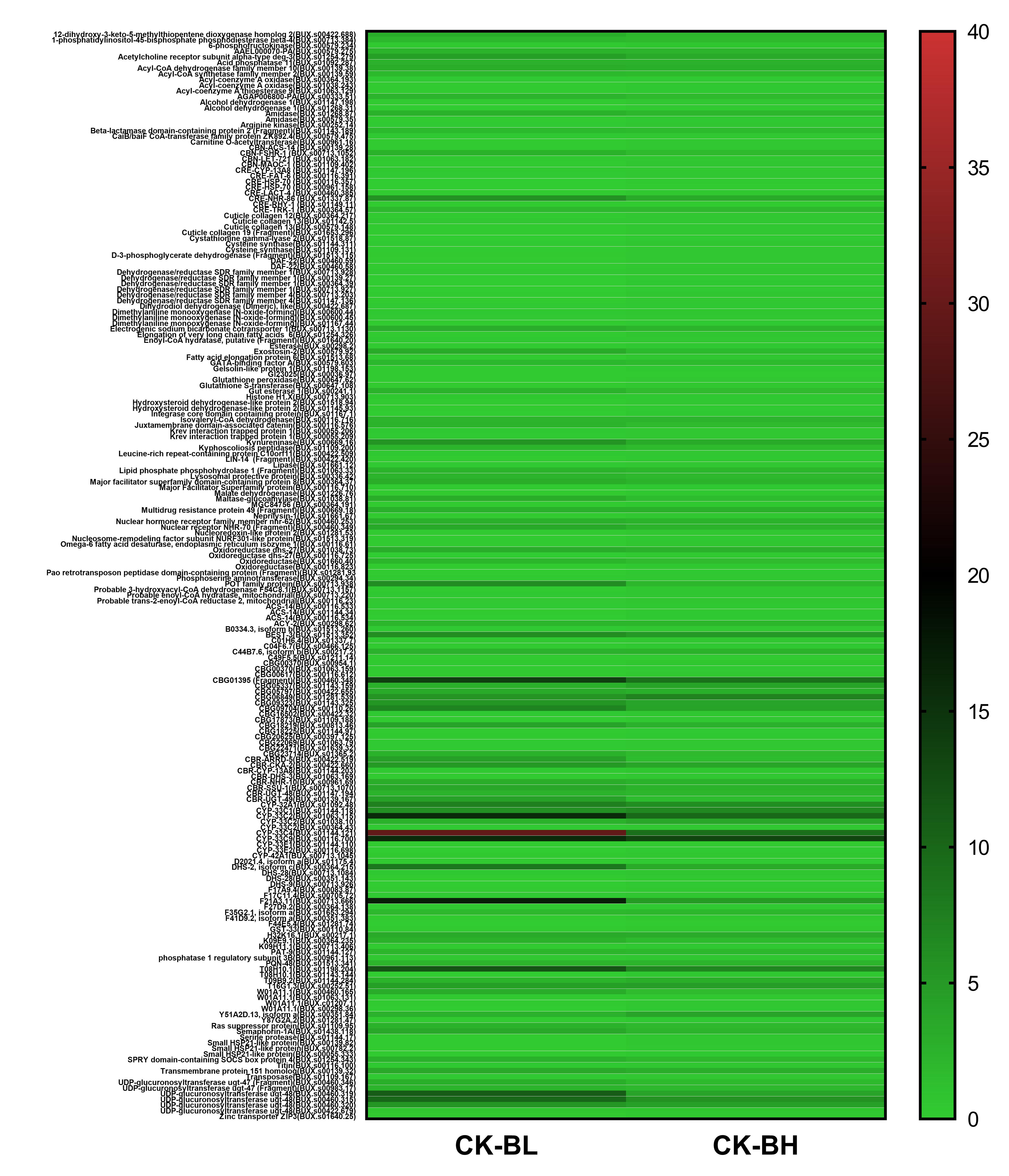

Supplement: Supplementary file 2 — Additional file 2: Figure S1. Heat map of the amplitude of 192 differentially expressed genes expressed in pine wood nematodes at high and low concentrations β-pinene conditions. [file 12864_2020_6876_MOESM2_ESM.tif]

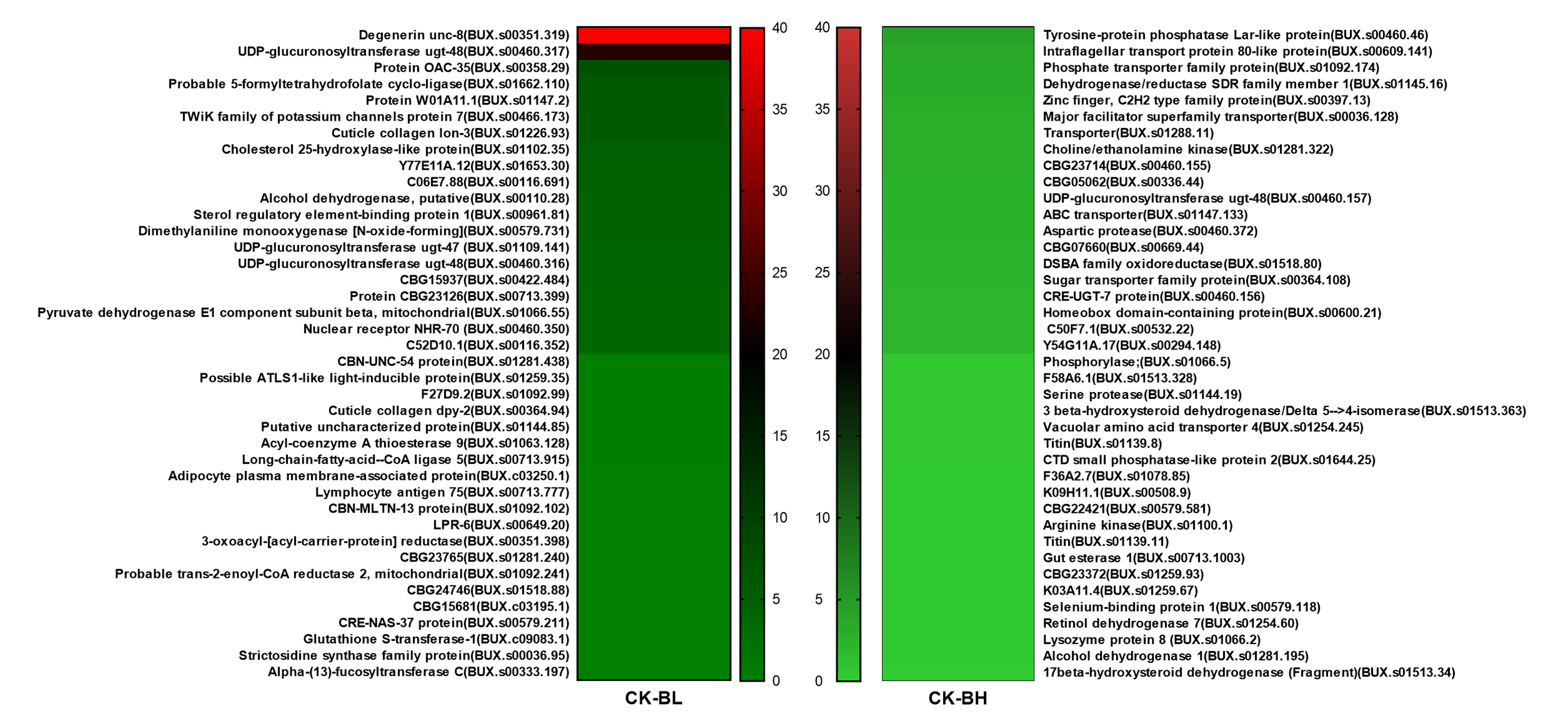

Supplement: Supplementary file 3 — Additional file 3: Figure S2. Heat maps of the top 20 differentially expressed genes for pine wood nematodes with high or low concentrations β-pinene treatment. [file 12864_2020_6876_MOESM3_ESM.tif]
